# HIV Prevalence and Risk Behaviors Among Mozambicans Working in South African Mines

**DOI:** 10.1007/s10461-014-0941-6

**Published:** 2014-11-15

**Authors:** Cynthia Semá Baltazar, Roberta Horth, Celso Inguane, Isabel Sathane, Freide César, Helena Ricardo, Carlos Botão, Ângelo Augusto, Laura Cooley, Beverly Cummings, Henry F. Raymond, Peter W. Young

**Affiliations:** 1 National Institute of Health, Instituto Nacional de Saúde, P.O. Box 264, Maputo, Mozambique; 2 University of California San Francisco (UCSF), San Francisco, CA USA; 3 International Training and Education Center for Health (I-TECH), Maputo, Mozambique; 4 Division of Global HIV/AIDS, Centers for Disease Control and Prevention (CDC), Atlanta, GA USA; 5 Division of Global HIV/AIDS, Centers for Disease Control and Prevention (CDC), Maputo, Mozambique

**Keywords:** Mineworkers, Mozambique, HIV, Prevalence, Risk behaviors, South African mines

## Abstract

Mineworkers are considered a population at risk for HIV due to risk behaviors associated with migratory work patterns. This was the first study in Mozambique to determine the prevalence of HIV and associated demographic and risk behaviors, and assess use and access to prevention and healthcare services among Mozambicans working in South African mines. Men who had worked in a South African mine in the past 12 months were recruited between February and May 2012 using time location sampling (TLS) at the Ressano Garcia border between Mozambique and South Africa. Demographic and behavioral data were collected through a standardized questionnaire, and HIV prevalence was estimated by testing dried blood spots (DBS) with two enzyme immunoassays. In total, 432 eligible mine workers were recruited. Mean age was 43 years. Most were married or cohabitating; among them, 12.6 % had two or more wives/marital partners in Mozambique. In the 12 months preceding the survey, 24.7 % had an occasional sexual partner, and 6.6 % had at least one partner who was a female sex worker. Only one in five (18.5 %) used a condom during last sex. HIV prevalence among mineworkers was 22.3 %, and 74.6 % of those who tested positive as part of the survey did not know their status. HIV prevalence was significantly higher (*p* = 0.018) among those that were uncircumcised (31.2 %) than those who were circumcised (18.5 %). Multiple partners (multiple spouses, cross-border relations, and multiple occasional partnerships), inconsistent condom use, and a high proportion of infected mineworkers who do not know their HIV status increases the risk of HIV transmission in this population. Combination strategies involving the promotion of condom use, HIV testing, and male circumcision should be strengthened among mineworkers.

## Introduction

For over 100 years Mozambican men have been migrating yearly to South Africa to work in the mining sector, living away from their home and family members [1]. Approximately 34,700 Mozambican men worked in South African mines in 2011.^1^ Men who stay away from their wives or girlfriends are more likely to have additional sexual partners and are therefore more likely to acquire HIV and other sexually transmitted infections (STIs) [2, 3].

HIV remains a major cause of morbidity and mortality worldwide, especially in sub-Saharan Africa [4]. Mozambique has the eighth highest HIV prevalence globally in the general population of 15–49 year olds, estimated at 11.1 % in 2012 [5]. The country has a predominantly heterosexual epidemic, with regional and provincial variations; HIV prevalence is highest in the South (17.8 %), followed by the Center (12.5 %), and is lowest in the North (5.6 %) [6]. Among the southern provinces in the country, where most mineworkers working in South Africa reside while in Mozambique, HIV prevalence among 15–49 year olds is highest in the provinces of Gaza (25.1 %), followed by Maputo and Maputo City (19.8 and 16.8 %, respectively), and is lowest in Inhambane (8.6 %) [6]. AIDS is the leading cause of adult deaths in Mozambique [4]. South Africa has one of the highest burdens of HIV infection in the world with an estimated 6.1 million people infected with HIV, and prevalence in adults (ages 15–49) was 17.9 % in 2012 [5].

Research on associations between migration and infection with HIV and other STI [2, 7, 8] has shown that, compared with the general population, migrant populations are at greater risk for HIV infection [2, 9, 10], because separation from their long-term sexual partners can lead them to seek other sexual partners [7, 11], as well as expose them to partners with higher risk of infection. Migration can also affect the behaviors of sex partners of migrants; due to the long absences of their primary sexual partners, migrants’ long-term sex partners may also engage in higher risk behaviors in the absence of their primary partner [2].

In Mozambique, data on HIV infection among populations at higher risk for HIV are limited, although the existence of these populations, including Mozambican mineworkers in South Africa, has been acknowledged in the third National Strategic Plan for HIV and AIDS Response [12]. To address this gap, in 2012, Mozambique conducted its first integrated biological and behavioral survey (IBBS) among Mozambicans working in South African mines. Its objectives included estimating the prevalence of HIV and associated risk behaviors, as well as assessing the use of and access to HIV prevention programs within this population in order to determine how to increase testing, treatment and prevention uptake in Mozambique and in South Africa. This paper presents the main findings of this IBBS.

## Methods

### Study Setting and Participants

Cross-sectional behavioral data reported in this analysis are from mineworkers recruited for interviews and HIV testing through time-location sampling (TLS) as part of an IBBS conducted in Mozambique in 2012. First, formative assessment, which precedes survey implementation, was used to identify locations associated with the population of interest. Then, potential days for sampling as well as potential time periods within those days, were identified. Investigators choose a random sample of day and time periods during which to recruit participants [13, 14].

In this case, formative assessment found that most Mozambican mineworkers are affiliated with TEBA Ltd., The Employment Bureau of Africa. TEBA Ltd. is a company established in 1902, to provide labor recruitment services to the Southern African mining Sector. Mozambican mineworkers must visit the TEBA Ltd. facilities in Ressano Garcia, a border town between Mozambique and South Africa, to sign or renew their contracts annually. At the same time, they must also receive a stamp at the local Mozambican Ministry of Labor (MITRAB) building, which is adjacent to TEBA Ltd. and connected directly by a doorway from TEBA Ltd. Information provided by TEBA Ltd. revealed that all contracts have a 12 month duration and are signed on a rolling basis throughout the calendar year; approximately 150 mineworkers have their contracts signed and stamped daily. Based upon this information, the decision was made to recruit participants using TLS at MITRAB facilities in Ressano Garcia (i.e., a single venue) just after having their contracts stamped. Miners recruited at TEBA Ltd. represented different mining industries, including gold, diamonds, coal, etc.

Sampling events consisted of 3 hour blocks during the days and hours when the offices of TEBA Ltd. and MITRAB in Ressano Garcia were open between February and May 2012. Fifty sampling events were randomly selected from Monday to Friday (7:30 am to 5:30 pm) to reach the expected survey sample size of 400 mineworkers. During sampling events, all potential participants were enumerated. As interviewers became available, potential participants were intercepted consecutively and invited to participate in the survey, until the sampling event ended.

Informed consent was sought from all who were intercepted and found eligible. To be eligible, a potential participant had to be a Mozambican man, be 18 years of age or older, have signed a contract the same day as interviews to work at a South African mine, and have greater than 12 months experience working in a South African mine. Eligible participants were asked to consent separately for the behavioral questionnaire and the preparation of dried blood spots (DBS) for HIV surveillance testing. All participants were also offered a rapid HIV test with pre- and post-test counseling and immediate results on-site. Participants were given a toiletries and HIV/STI prevention kit as a compensation for their time worth ~8 USD.

### Behavioral Questionnaire

The behavioral questionnaire was adapted from questionnaires from similar surveys in sub-Saharan Africa and included core HIV prevention indicators (FHI Guidelines, UNAIDS core indicator guidelines). Computer-assisted personal interviews (CAPI) were conducted privately on location permitting interviewers to enter responses directly into a netbook. The survey tool included a sexual matrix with detailed information on sexual behaviors with last three sexual partners.

### Sample Collection and Testing

After the interview, participants were sent to a separate room where they received HIV counseling and provided a blood sample by finger prick for rapid HIV testing done on-site with immediate return of results. Screening was done with Determine™ HIV-1/2 (Alere, USA), and those preliminary positive were confirmed with Uni-Gold™ *HIV* (Trinity Biotech, Ireland) following national guidelines for HIV testing. Dried blood spots were collected from all consenting participants. HIV prevalence estimates in this paper were based on EIA testing conducted at the national laboratory using these DBS samples. Participation in the survey was anonymous; no personal identifiers were collected. Questionnaires and specimens were coded with a study number. These tests were used solely for surveillance purposes and were not returned to individual participants. Screening was done with Vironostika HIV Uniform II plus O (Biomerieux SA, France). Reactive samples and 5 % of the negative samples were confirmed using Murex HIV 1.2.O (Murex Biotech Limited, UK). Discrepant results were retested using Genscreen HIV 1/2 Version 2 (Bio-Rad, France).

### Data Analysis

Only one venue was identified for recruitment, thus venue attendance did not vary by participants. Therefore analyses for this paper are unweighted.

Data analysis was conducted using R software (Version, 2.13, R Foundation for Statistical Computing, Austria). The prevalence of HIV is reported with 95 % confidence intervals (CIs). The association between HIV infection and other variables was analyzed using Pearson’s *χ* ^2^test and Fisher’s exact test, for which *p* values are presented. No multivariate analysis was conducted.

### Ethical Considerations

This survey protocol was approved by the Mozambican National Bioethics Committee for Health, and the Institutional Review Board of the University of California at San Francisco, and was reviewed by the CDC Division of Global HIV/AIDS Program as non-engaged research.

## Results

In total, 727 potential participants were intercepted, 709 (98 %) of whom were eligible for the survey. Of all eligible potential participants, 430 (61 %) agreed to participate and completed the survey questionnaire (enrollment continued after reaching the target of 400 due to a higher than anticipated percent of refusal for DBS). Among those who agreed to participate, 323 consented to preparation of DBS (75 % who completed the survey). Among those who did not consent to preparation of DBS, most did not provide a specific reason for not doing so (*n* = 35), another 31 said they did not want or feared a finger prick, 17 had recently given blood for other purposes, 13 had no time and 11 gave some other reason.

### Demographic Characteristics of Mineworkers

The general characteristics of mineworkers are shown in Table 1. Age ranged from 23 to 68 with an average of 43 years; the majority was older than 30 years of age. The main language spoken at home by mineworkers was Xichangana/Xironga (54.0 %), followed by Xitswa (19.5 %). About half of mineworkers were Protestant or Evangelical (49.5 %). Miners reported their residence for both Mozambique and South Africa. The largest percentage of mineworkers reported their main residence in Mozambique to be in the province of Gaza (42.3 %), followed by Maputo (35.8 %) and Inhambane (19.3 %). In South Africa, the main provinces of residence were North West (57.7 %) and Gauteng (19.3 %) (Table 1).

**Table 1:** Socio-demographic characteristics of mineworkers, Mozambique, 2012

Characteristics	*N* = 430	% (CI 95 %)
Age		
21–30	28	6.5 (4.2–8.8)
31–40	144	33.5 (29.0–37.9)
41–50	132	30.7 (26.3–35.1)
≥51	126	29.3 (25.0–33.6)
Primarily language		
Xichangana/Xironga	232	54.0 (49.2–58.7)
Xitswa	101	19.5 (19.5–27.5)
Chope	58	13.5 (10.3–16.7)
Portuguese	19	4.4 (2.5–6.4)
Other	20	4.7 (2.7–6.6)
Religion		
Catholic	94	21.9 (18.0–25.8)
Protestant/evangelic	213	49.5 (44.8–54.3)
Zion	83	19.3 (15.6–23.0)
Other	40	9.3 (6.6–12.0)
Circumcised		
Yes	287	66.7 (62.3–71.2)
No	143	33.3 (28.8–37.7)
Province of main residence in Mozambique		
Inhambane	83	19.3 (15.6–23.0)
Gaza	182	42.3 (37.7–47.0)
Maputo province/city	154	35.8 (31.3–40.3)
Other	11	2.6 (1.1–4.1)
Province of main residence in South Africa		
North West	248	57.7 (53.0–62.3)
Gauteng	83	19.3 (15.6–23.0)
Free State	36	8.4 (5.8–11.0)
Limpopo	29	6.7 (4.4–9.1)
Other	34	7.9 (5.4–10.5)
Education		
No formal education	25	5.8 (3.6–8.0)
Some primary/Literacy	341	79.3 (75.5–83.1)
Some secondary or greater	64	14.9 (11.5–18.2)
Main occupation in the mine		
Engine driver, pump operator, crane operator, mechanic	166	38.6 (34.0–43.2)
Miscellaneous work	63	14.7 (11.3–18.0)
Perfurator, plumber, electrician, excavator, assembler	88	20.5 (16.7–24.3)
Foremen, supervisor	78	18.1 (14.5–21.8)
Other	35	8.1 (5.6–10.7)
Years worked in South African mines		
1–9	80	18.6 (14.9–22.3)
10–19	204	47.4 (42.7–52.2)
20–29	104	24.2 (20.1–28.2)
≥30	42	9.8 (7.0–12.6)
Place of lodging in South Africa		
Hostel	177	41.2 (36.5–45.8)
With relatives	84	19.5 (15.8–23.3)
With girlfriend/lover/friend	7	1.6 (0.8–2.8)
With a friend/colleague	36	8.4 (5.8–11.0)
Alone	126	29.3 (25.0–33.6)

More than three quarters (79.3 %) of mineworkers had attended some primary school or some literacy classes, and 14.9 % had attended some secondary school or higher. Most (38.6 %) were primarily working in the mines as engine drivers, pump operators, crane operators, or mechanics. Approximately 81 % of participants had 10 or more years of experience working in the mines. Mineworkers mostly lived in hostels (41.2 %); another 19.5 % resided with relatives and nearly one third lived alone (29.3 %).

### Marital Status, Sexual History and Risk Behavior

The majority of mineworkers (96.3 %) were married or cohabitating. Among married mineworkers, 99 % had at least one wife in Mozambique and 9.2 % had at least one wife in South Africa; 8.2 % had at least one wife in each country (Table 2).

**Table 2:** Marital status and sexual history of mineworkers, Mozambique, 2012

Characteristics	*N* = 430	% (CI 95%)
Marital status of mineworkers		
Never married	3	0.7 (0.0–1.5)
Married/cohabitating	414	96.3 (94.5–98.1)
Windower/divorced/separated	13	3.0 (1.4–14.6)
Number of wives or conjugal partners in Mozambique		
0	4	1.0 (0.0–1.9)
1	358	86.5 (83.2–89.8)
≥2	52	12.6 (9.4–15.8)
Number of wives or conjugal partners in South Africa		
0	376	90.8 (88.0–93.6)
1	36	8.7 (6.0–11.4)
≥2	2	0.5 (0.0–1.2)
Number of wives or conjugal partners in both countries		
1 wife living in South Africa	4	1.0 (0.0–1.9)
1 wife living in Mozambique	328	79.2 (75.3–83.1)
>1 wife living in Mozambique	48	11.6 (8.5–14.7)
At least 1 wife in each country	34	8.2 (5.6–10.9)
Age at sexual debut with a woman (missing = 16)		
<18	113	27.3 (23.0–31.6)
18–19	148	35.7 (31.1–40.4)
20–24	127	30.7 (26.2–35.1)
≥25	26	6.3 (3.9–8.6)
Sexual partners, last 12 months (missing = 3)		
0	4	0.9 (0.0–1.9)
1	204	47.8 (43.0–52.5)
2	142	33.3 (28.8–37.7)
≥3	77	18.0 (14.4–21.7)
Main sexual partners (girlfriends or wives), last 12 months (missing = 6)		
0	7	1.7 (0.4–2.9)
1	352	83.0 (79.4–86.6)
2	61	14.4 (11.0–17.7)
≥3	4	0.9 (0.0–1.9)
Occasional sexual partners, last 12 months (missing = 7)		
0	264	62.4 (57.8–67.0)
1	103	24.3 (20.3–28.4)
2	41	9.7 (6.9–12.5)
≥3	15	3.5 (1.8–5.3)
Paid sexual partners, last 12 months (missing = 5)		
0	397	93.4 (91.1–95.8)
≥1	28	6.6 (4.2–8.9)

Two or more partners in the past 12 months were reported by 51.3 % of participants. Most (93.4 %) reported that they did not pay for sex in the last 12 months. No mineworkers reported having ever engaged in sexual intercourse with a man.

In the 12 months preceding the survey, 18.5 % of mineworkers used a condom the last time they had sex (Table 3). Among those who had at least one occasional or transactional sexual partner in the last 12 months, 51.8 % used condoms at last sex with all occasional or transactional partners in the same period, and among those with a spouse or main partner in the last 12 months, 13.6 % used condoms at last sex with all spouses or main partners in the 12 months preceding the survey.

**Table 3:** Condom use, access to HIV and STI prevention and healthcare services, and HIV testing history among mineworkers, Mozambique, 2012

Characteristics	*N* = 430	% (95 % CI)
Used a condom the last time they had sex in the last 12 months^a^		
Yes	78	18.5 (14.8–22.2)
No	343	81.5 (77.8–85.2)
Used a condom the last time they had sex with each spousal partner in the last 12 months^a^		
Yes	56	13.6 (10.3–16.9)
No	356	86.4 (83.1–89.7)
Used a condom the last time they had sex with each occasional or transactional sex partner in the last 12 months^b^		
Yes	71	51.8 (43.5–60.2)
No	66	48.2 (39.8–56.5)
Had access to free condoms in the last mine where they worked in the last 12 months		
Yes	404	94.0 (91.7–96.2)
No	26	6.0 (3.8–8.3)
Participated in educational sessions on HIV in the last 12 months		
Yes	342	79.5 (75.7–83.3)
No	88	20.5 (16.7–24.3)
Received condoms, lubricants or HIV prevention leaflets in the last 12 months		
Yes	301	70.0 (65.7–74.3)
No	129	30.0 (25.7–34.3)
Had any unusual discharge, sore or ulcer on the penis or was informed they might have an STI in the last 12 months		
Yes	35	8.1 (5.6–10.7)
No	395	91.9 (89.3–94.4)
Had ever been tested for HIV		
Yes	382	88.8 (85.9–91.8)
No	48	11.2 (8.2–14.1)
Date of most recent HIV test^c^ (missing = 1)		
≤12 months	326	85.6 (82.0–89.1)
>12 months	55	14.4 (10.9–18)
Result of the most recent HIV test^c^ (missing = 9)		
Positive	27	7.2 (4.6–9.9)
Negative	345	92.5 (89.8–95.2)
Indeterminate	1	0.3 (0.0–0.8)
Perception of risk of HIV infection^d^ (missing = 15)		
No risk	51	13.1 (9.8–16.5)
Low risk	93	24.1 (19.7–28.2)
Moderate risk	139	35.8 (31.1–40.6)
High risk	105	27.1 (22.6–31.5)

^a^Includes only mineworkers who had sex with a spousal partner of their last three partners in the last 12 months

^b^Includes only mineworkers who had an occasional or transactional sex partner of their last three partners in the last 12 months

^c^Subset of 382 mineworkers who had ever been tested for HIV

^d^Subset of mineworkers who had never received an HIV-positive test result before the survey

### Access to and Use of Healthcare Services and Prevention Programs

In the last mine where they worked, 94.0 % of the mineworkers had access to free condoms (Table 3). In the 12 months preceding the survey, 79.5 % of the mineworkers participated in educational sessions about HIV. Within the same period, 70.0 % of the mineworkers received condoms, lubricants or HIV prevention pamphlets.

In the 12 months preceding the survey, 8.1 % of the mineworkers reported having had symptoms or having been informed that they might have an STI. Among them, 31.6 % did not seek medical advice or treatment (not shown in table).

### Reported HIV Testing History and HIV Risk Perception

Mineworkers were asked to report prior HIV testing history and perceived risk. Approximately nine out of every 10 mineworkers (88.8 %) reported ever having tested for HIV (Table 3). Among those who had ever tested, 85.6 % tested within the 12 months preceding the survey, of which 92.5 % tested negative and 7.2 % reported that they tested positive at their most recent HIV test. Participants were asked to provide the principal reasons (multiple response question) for having had their last HIV test. The majority (74.0 %) reported testing because of their desire to know their status, 13.1 % tested at the request of the employer and 11.5 % were advised to test by a healthcare worker (not shown).

Among those who had never tested positive for HIV, 35.8 % believed that their risk of HIV infection was moderate and 27.1 % believed they were at a high risk of HIV infection. Nearly all (99.7 %) believed their HIV status at the time of the survey was negative.

### HIV Prevalence in Mineworkers

HIV prevalence was estimated among 318 (74.0 %) men who provided DBS specimens. Overall HIV prevalence was 22.3 % (95 % CI 17.8–26.9). The 23–30 age group had an HIV prevalence of 13.6 % (95 % CI 0.0–28.0); the 31–40 age group had the highest HIV prevalence of 26.7 % (95 % CI 18.2–35.1); the 41–50 age group had an HIV prevalence of 20.6 % (95 % CI 12.6–28.7); and the 51 and older age group had an HIV prevalence of 21.3 % (95 % CI 13.0–29.6) (Table 4).

**Table 4:** HIV prevalence among mineworkers by socio-demographic characteristics, Mozambique, 2013 (*N* = 318)

Characteristic	*n*/*N* ^a^	Prevalence	(95 % CI)	*p* value
HIV Prevalence (missing^b^ = 112)	71/318	22.3	(17.8–26.9)	
Age group				
23–30	3/22	13.6	(0.0–28.0)	0.561
31–40	28/105	26.7	(18.2–35.1)	
41–50	20/97	20.6	(12.6–28.7)	
≥51	20/94	21.3	(13.0–29.6)	
Primary language spoken at home				
XiChangana/Xironga	48/170	28.2	(21.5–35.0)	<0.001
Xitswa	11/72	15.3	(7.0–23.6)	
Chope	2/48	4.2	(0.0–9.8)	
Portuguese/other	10/28	35.7	(18.0–53.5)	
Religion				
Catholic	13/64	20.3	(10.5–30.2)	0.158
Protestant/Evangelic	34/167	20.4	(14.3–26.5)	
Zion	20/60	33.3	(21.4–45.3)	
Other/None	4/27	14.8	(1.4–28.2)	
Wives or conjugal partners				
1	55/263	20.9	(16.0–25.8)	0.527
≥2	10/37	27.0	(12.7–41.3)	
Circumcised				
Yes	41/222	18.5	(13.4–23.6)	0.018
No	30/96	31.2	(22.0–40.5)	
Education				
Less than secondary	63/275	22.9	(17.9–27.9)	0.665
Secondary or higher	8/43	18.6	(7.0–30.2)	
Province of residence, Mozambique				
Maputo province/city	17/62	27.4	(16.3–38.5)	0.062
Gaza	35/134	26.1	(18.7–33.6)	
Inhambane	17/116	14.7	(8.2–21.1)	
Province of residence, South Africa				
North West	40/184	21.7	(15.8–27.7)	0.602
Gauteng or free state	23/90	25.6	(16.5–34.6)	
Other	8/44	18.2	(6.8–29.6)	
Years worked in South Africa				
1–9	12/64	18.8	(9.2–28.3)	0.744
10–25	48/207	23.2	(17.4–28.9)	
≥25	11/47	23.4	(11.3–35.5)	
Annual trips back to Mozambique, last 12 months				
1–3	40/166	24.1	(17.6–30.6)	0.854
≥4	20/90	22.2	(13.6–30.8)	

^a^Numerator = HIV positive participants and denominator = total number of participants in each category

^b^Missing HIV test results are due to refusal to provide dried blood spots for HIV testing

HIV prevalence was higher among mineworkers with province of residence in Maputo (27.4 %; 95 % CI 16.3–38.5), and Gaza (26.1 %, 95 % CI 18.7–33.6), followed by Inhambane (14.7 %, 95 % CI 8.2–21.1), however this difference was not statistically significant. No differences were found in HIV prevalence by education level, religion, province of residence in South Africa, years of work in South Africa, or number of annual trips back to Mozambique.

Nearly three quarters (74.6 %) of HIV-positive mineworkers were not aware of their status. Among mineworkers who reported a negative HIV test in the 12 months preceding the survey, 14.5 % tested positive for HIV in the survey (Table 5). Of the 27 participants who self-reported being HIV-positive, 21 (77.8 %) were on antiretroviral therapy.

**Table 5:** Knowledge of HIV serostatus among mineworkers testing HIV positive during the survey (*N* = 71) and HIV test results among mineworkers who reported an HIV negative test in the 12 months preceding the survey (*N* = 299)

Characteristic	*N*	% (95 % CI)
Knowledge of HIV serostatus^a^		
Never diagnosed as HIV+	53	74.6 (64.5–84.8)
Previously diagnosed as HIV+	18	25.4 (15.2–35.5)
Current HIV serostatus^b^ (missing = 78)		
HIV negative	189	85.5 (80.9–90.2)
HIV positive	32	14.5 (9.8–19.1)

^a^Subset of mineworkers who tested positive for HIV in the survey

^b^Subset of mineworkers who were tested for HIV in the 12 months preceding the survey and had a negative result

### Circumcision

Two thirds of mineworkers (66.7 %) were circumcised. HIV prevalence among uncircumcised mineworkers (31.2 %) was significantly higher than among circumcised mineworkers (18.5 %) (Table 4). The percentage of mineworkers that were circumcised was highest in Inhambane province 96.1 % (95 % CI 93.0–99.1) (not shown), where the prevalence of HIV among mineworkers was the lowest at 14.7 % (95 % CI 8.2–21.1).

## Discussion

The prevalence of HIV among Mozambican mineworkers working in South Africa (22.3 %, 95 % CI 17.8–26.9) was high and contrasts sharply with that in the general male Mozambican population age 15–49 years of age (9.2 %, 95 % CI 4.6–13.9) [6]. However, HIV prevalence in this survey does not include retired mineworkers or those who may be unable to work due to illness, such as with advanced HIV disease. As such, true prevalence among mineworkers in Mozambique may be even higher than what was documented as part of this study, reflecting the healthy worker effect [15].

Although HIV prevalence is seemingly higher among mineworkers working in South Africa than the general adult Mozambican male population [6], their higher prevalence may in part be explained by older average age and residence in the southern region of Mozambique, which has the highest HIV prevalence in the country. Nonetheless, the magnitude of HIV prevalence coupled with frequent risky sexual practices (e.g. more than half of men in this study had two or more partners in the past 12 months and only about one in ten used a condom at last sex with a stable partner) put mineworkers at great risk of HIV acquisition and transmission. Additionally, the amplitude of the HIV epidemic among mineworkers can decrease productivity, affect the families of the infected workers, and strain both the South African and the Mozambican healthcare systems [2].

HIV prevalence varied significantly by primary language spoken at home. Primary home language is strongly associated with the geographical region of residence in Mozambique. As such, understanding the primary languages used by mineworkers is useful in planning HIV prevention and care and treatment messages.

Mining areas often do not provide accommodation for spouses or families, and most mineworkers migrate alone. As a result, and because mineworkers earn higher incomes than the average male in Mozambique, they may choose to have, and be able to support, wives in both Mozambique and South Africa, or may interact with female sex workers in the areas surrounding mines [8]. In this survey, 8.2 % of the participants had at least one wife in both Mozambique and South Africa. And, while only 6.6 % of mineworkers reported they had paid for sex in the last 12 months, nearly 40 % of mineworkers had an occasional sex partner in that same time period; and, condoms were used by merely 50 % of mineworkers with such partners.

Condom use was generally low among mineworkers in this study, despite the fact that nine out of every 10 mineworkers in the study reported access to free condoms in the last mine where they worked in the preceding 12 months.

HIV prevalence among non-circumcised mineworkers was higher than circumcised mineworkers, which is consistent with landmark studies that showed that male circumcision lowers the risk of acquiring HIV [16–18]. Overall HIV prevalence was lowest among participants from Inhambane, where the percentage of participants who have been circumcised is the greatest.

The percentage of mineworkers who reported recently having been tested for HIV (past 12 months) is exceptionally high (75.8 %) relative to the uptake of HIV testing across sub-Saharan Africa and Mozambique [19]. Yet, 74.6 % of HIV-positive mineworkers in this study were not aware of their HIV positive status. There may be several reasons for this discrepancy. Social desirability bias may account for an over-reporting of recent HIV testing. Additionally, mineworkers may feel pressured to undergo HIV testing by their employers; admitting non-adherence could have negative ramifications. Also, individuals with known HIV infection may be hesitant to disclose their HIV status due to HIV stigma, and thus past positive results may be under-reported. The proportion of newly diagnosed HIV infections that are attributable to incident infection is not currently known. Regardless, HIV testing targeted towards populations at higher risk for HIV infection is an evidence-based, cost-effective, and scalable intervention; HIV testing and diagnosis can lead to initiation of antiretroviral treatment and can promote behavioral change, both of which can optimize clinical outcomes and reduce the risk of HIV transmission to others [20]. Efforts should be continued to ensure complete access to and uptake of HIV testing.

There are several limitations to this study. Participants were recruited between February and May, and may not represent all mineworkers recruited by TEBA Ltd. year round. Also the study recruited only mineworkers hired by TEBA Ltd., although this company is recruits most of the Mozambican mineworkers hired in South Africa. Data were self-reported and are subject to social-desirability and recall bias. Additionally, of all eligible mineworkers approached to participate in the study, 61.0 % agreed to participate, of which 75.0 % agreed to provide a DBS sample for HIV testing. This response rate may have introduced selection bias and may limit the generalizability of the findings. Finally, this survey targeted mineworkers renewing their contract to return to the mines, and may not represent mineworkers who abandoned mining work, those who have retired, or who are not currently in the work force due to health-related reasons such as HIV and TB, introducing a possible healthy worker effect. However, since the vast majority of contracts (99 %) are processed through a single facility at the border of South Africa and Mozambique, we believe our sample is representative of Mozambican men working in South African mines and is free from many of the limitations of traditional TLS surveys, where the frequency of attendance at venues must be established, estimated, or assumed.

In conclusion, this study documented a high prevalence of HIV and low awareness of HIV status along with high prevalence of sexual risk behaviors among Mozambican men who migrate to South Africa to work in the mining sector. Low awareness of HIV-positive status, multiple sex partners and low condom use can increase the risk of HIV transmission. Mathematical models suggest HIV transmission in populations with high levels of migration coupled with risk behaviors in rural areas among spouses of migrants as well as among non-migrant populations may contribute to the continuing high HIV prevalence in the southern provinces of Mozambique [21]. This IBBS survey contributes to the small but growing body of literature regarding mineworkers. HIV prevalence, associated risk factors, and access to prevention and healthcare services among Mozambican mineworkers should continue to be monitored. Although more difficult to implement, surveillance of HIV and associated risk behaviors among non-migrants in areas with high levels of oscillating migration would help inform prevention strategies. Combination strategies involving the promotion of condom availability and use, HIV prevention counseling and education in local languages, HIV testing, and male circumcision should be strengthened in Mozambique, where mineworkers reside when not working in South Africa [22, 23].

## Footnotes

1 Communication from The Employment Bureau of Africa (TEBA) Ltd, June, 2012.
